# Implementation and evaluation of a multidisciplinary training course in waiting list management

**DOI:** 10.1590/0034-7167-2025-0142

**Published:** 2025-12-08

**Authors:** Rosa Ladi Lisbôa, Adriana Aparecida Paz

**Affiliations:** I Hospital Nossa Senhora da Conceição. Porto Alegre, Rio Grande do Sul, Brazil; II Universidade Federal de Ciências da Saúde de Porto Alegre. Porto Alegre, Rio Grande do Sul, Brazil

**Keywords:** Health Management, Waiting Lists, Educational Technology, Technology Applied to Waiting Lists, Nursing., Gestión em Salud, Listas de Espera, Tecnología Educacional, Tecnologia Aplicada a las Listas de Espera, Enfermería.

## Abstract

**Objective:**

To implement and evaluate satisfaction and knowledge retention in a multidisciplinary training course on waiting list management.

**Method:**

This methodological study involved two phases of the ADDIE instructional design model. The course was delivered through a virtual learning environment (VLE) between October 2023 and June 2024. Data were collected using questionnaires and VLE-generated reports and were analyzed using descriptive statistics and the Wilcoxon test (p<0.05).

**Results:**

A total of 768 healthcare professionals enrolled in the course; 429 (55.9%) accessed the VLE, and 255 (59.4%) completed the course. Course evaluation showed high satisfaction levels with the presentation, content, and applicability to professional practice. The analysis revealed significant participant knowledge retention (z = -8.09, p < 0.001).

**Conclusion:**

The findings demonstrate the course’s positive impact on participant training, as reflected in the high satisfaction ratings and significant knowledge retention.

## RESUMEN

**Objetivo:**

Implementar y evaluar la satisfacción y la retención de conocimientos en un curso de formación multidisciplinaria sobre gestión de listas de espera.

**Método:**

Estudio metodológico desarrollado en dos fases del modelo ADDIE, implementado en un ambiente virtual de aprendizaje (AVA) entre octubre de 2023 y junio de 2024. Los datos se recopilaron mediante cuestionarios y reportes del AVA, y se analizaron con estadística descriptiva y la prueba de Wilcoxon (p < 0,05).

**Resultados:**

Participaron 768 profesionales de la salud; 429 (55,9 %) accedieron al AVA y 255 (59,4%) completaron el curso. La evaluación indicó alta satisfacción con el diseño, los contenidos y su aplicabilidad. Se observó una retención significativa de conocimientos entre los participantes (z = -8,09; p < 0,001).

**Conclusión:**

Los resultados evidencian el impacto positivo del curso en la formación profesional, reflejado en la retención del aprendizaje y en la valoración positiva de los participantes.

## INTRODUCTION

Waiting lists for surgical and clinical patients represent a nationwide challenge, generating negative impacts not only for healthcare institutions and the broader system but especially for patients awaiting care. Delays in performing these procedures can lead to several complications for patients, including the deterioration of health conditions, prolonged physical and emotional suffering, loss of bodily function, and even death^(1,2)^. There are also economic consequences, as patients with more severe clinical conditions tend to remain hospitalized longer, requiring greater resource use and facing increased exposure to adverse events such as hospital-acquired infections-creating a vicious cycle of extended hospital stays and heightened risk^(3,4)^.

Two issues stand out among the current shortcomings of healthcare systems: the management of elective surgery waiting lists and the insufficient professional training of healthcare staff for list regulation. In this context, continuing education for the health workforce is essential-an imperative enshrined in the Brazilian Constitution^(5)^.

Technological advancements in education have shown that learning is no longer confined to traditional classroom settings. This shift reinforces the need to develop pedagogical practices that expand teaching and learning opportunities in cyberspace, with diverse learning paths, contexts, and learner profiles^(6)^.

In professional training, distance education (DE) emphasizes knowledge construction and sharing, putting educational principles and goals into practice. Regardless of time or place, individuals can take ownership of their learning through diverse educational materials and communication tools that support synchronous or asynchronous interactivity and collaborative learning^(7)^.

This study is based on a professional master’s thesis that developed an educational technology designed, evaluated, and implemented with scientific and technical rigor. The technology was structured as a training course aimed at qualifying healthcare professionals and administrative staff working in regulating waiting lists for elective surgical and clinical care^(5)^.

Optimizing the management of elective surgery waiting lists is essential for the effective functioning of Brazil’s Unified Health System (SUS). In this regard, the educational technology developed in this study plays a relevant role by contributing to training the multiple professional categories involved in managing these waiting lists.

## OBJECTIVE

To implement and evaluate satisfaction and knowledge retention in a multidisciplinary training course on waiting list management.

## METHODS

### Ethical aspects

The data analyzed in this study come from a master’s dissertation^(8)^.The study was approved by the Research Ethics Committee and complied with regulatory guidelines and standards for research involving human subjects. The Informed Consent Form (ICF) was obtained from all participants through the Informed Consent Registration (ICR). The document guaranteed anonymity, confidentiality, privacy, and the protection of personal information. The project received ethics approval under opinion no. 5.103.774/2021.

### Study design, period, and setting

This methodological study was developed in five stages, based on the ADDIE instructional design model (an acronym for Analysis, Design, Development, Implementation, and Evaluation)^(8)^. The model is divided into two phases: design and implementation. The design phase (Analysis, Design, and Development) encompassed the development of an educational technology in the format of a multidisciplinary training course. During this phase, a demand analysis, pedagogical course design, and content development in storyboards were conducted, followed by a content adequacy review by a committee of experts. This phase of the research was recently published^(5)^. The implementation phase included the deployment of the course in a virtual learning environment (VLE), as well as the evaluation of participant satisfaction and knowledge retention. These last two steps completed the ADDIE model cycle and are the focus of this study.

The training product was developed and implemented within a VLE as part of a Professional Master’s Program in Nursing at a public university in southern Brazil. The implementation team included two nurses (one PhD and one MSc) and two undergraduate nursing students (research and teaching initiation scholarship holders). The phases were carried out between October 2023 and June 2024.

### Methodological procedures

#### First stage: Implementation of the course as an educational technology

The course was implemented following the design phase under the ADDIE model. The storyboards developed in PowerPoint^®^ were exported to the Articulate Storyline 3^®^ software, which enabled the creation of the course interface in the virtual classroom, incorporating various graphic elements and responsive design features adapted to different screen sizes and resolutions. Additionally, the platform offered optimized solutions for touchscreen devices, commonly used in mobile access.

**Figure 1:**
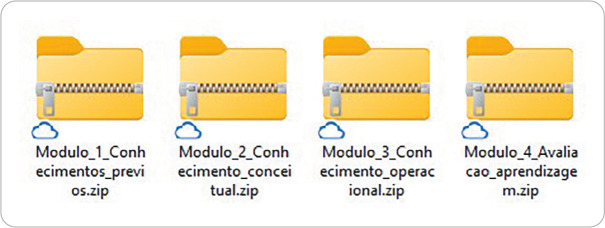
Course SCORM package. Porto Alegre, Rio Grande do Sul, Brazil, 2024

The Sharable Content Object Reference Model (SCORM) is a file package generated by exporting content from Articulate Storyline 3^®^ in its native format (*.STORY). SCORM contains technical information and parameters compatible with Learning Management Systems (LMS) within a VLE. This standard format is widely used in distance education and allows seamless content integration into LMS platforms, as illustrated in Figure 1.

The virtual classroom was hosted by the university’s Distance Education and Information Technology Centers, which enabled the structuring and organization of learning objects in the VLE (https://moodle.ufcspa.edu.br/course/view.php?id=2768). Custom layouts, texts, and instructional videos were developed based on the storyboard’s visual identity to facilitate participant access and engagement.

The course was registered in the university’s Unified Registration System (SIUR) in December 2023 for review by the Graduate Programs Committee. Approval was granted for offering 1,000 slots to both the internal academic community and external participants (https://bit.ly/GestaoLeitosPPGEnf), with enrollment open from December 2023 to March 2024.

Outreach was conducted through the social media platforms of the TeGEST Research Group (Technology, Management, Education, and Occupational Safety), part of the university’s Graduate Program in Nursing, as well as through the official channels of the Federal and Regional Nursing Councils. Email invitations and registration forms were also sent to higher education institutions, healthcare facilities, professional boards, and nursing and medical associations. The self-instructional course was delivered in a DE format from January to April 2024, with a total workload of 20 hours.

#### Second stage: Evaluation of participant satisfaction and knowledge retention in the multidisciplinary training course

**Figure 2:**
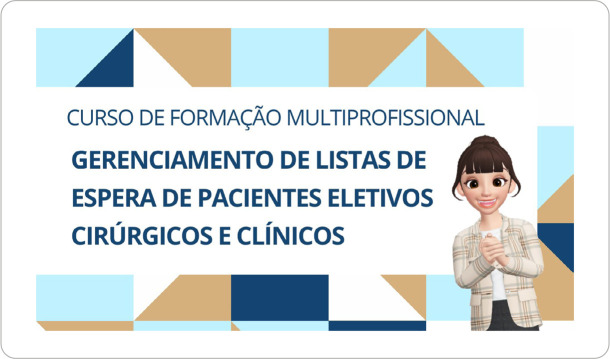
Multidisciplinary training course on the management of waiting lists for elective surgical and clinical patients. Porto Alegre, Rio Grande do Sul, Brazil, 2024

This was an exploratory-descriptive study with a quantitative approach. The study setting was the “Multidisciplinary Training Course on the Management of Waiting Lists for Elective Surgical and Clinical Patients” (Figure 2), with a total workload of 20 hours, made available in the institution’s VLE.

Modules 1 and 4 shared a similar structure. The first included a diagnostic activity (prior to the content) titled “Prior knowledge of waiting list management for elective patients,” while the last presented an evaluative activity (following the content) titled “Learning assessment on waiting list management for elective patients.” Each module had an estimated duration of one hour and featured three clinical cases, each containing seven single-choice questions with four answer options.

A clickable interface allowed participants to select the clinical case they wished to complete based on their interests. Various strategies were used in designing the questions to ensure variation in cognitive demand and complexity. The two modules differed in the sequence of clinical cases, fictional names, geographic context, ordering of correct answers, and the inclusion of feedback at the end of each question, which was provided only in the evaluative activity.

A total of 428 participants agreed to take part in the course satisfaction assessment (presentation, content, and applicability), conducted through a questionnaire available in the final module. Participants also provided informed consent by completing the ICF available in the VLE. This study included only those who completed all four course modules-ensuring full access to the content and interactivity offered by the DE format-and who completed the evaluation questionnaire. After formalizing consent via the ICR, an additional questionnaire was made available to collect information on how participants heard about the course and their geographic origin. Based on participants’ performance in Modules 1 and 4, data on knowledge retention were extracted from the VLE.

Data was collected exclusively using two sources: the databases from Google Forms^®^ and the participant activity report generated by the VLE. Information regarding enrollment, dropouts, and course completion was collected from the VLE through a structured data extraction report.

In June 2024, data from the satisfaction assessment were extracted from the Google Forms^®^ database of participants who completed the “Professional Training Course Evaluation” questionnaire.

The questionnaire to assess participant satisfaction included 26 items (multiple-choice, open-ended single-choice, and numerical scale questions). It was organized into four sections: (a) Presentation, (b) Content, (c) Applicability, and (d) Sociodemographic and professional background. The questions addressed the quality of the instructional methods and technological tools used, the clarity and coherence of the assessment items, the appropriateness of the content and language, the applicability and relevance to professional practice, and the course’s ability to spark interest and meet expectations. These aspects were assessed using dichotomous questions (yes or no), a five-point Likert scale, and a 0-100-point numerical scale, based on a previous study^(9)^ and adapted by the authors.

### Data analysis and statistics

The data were extracted from the VLE database in a Microsoft Excel^®^ spreadsheet containing participant consent records and responses collected through the Google Forms^®^ questionnaire. The files were saved locally in Excel^®^ format to ensure data security and prevent participant identification^(10)^. The database included only the variables relevant to this study, and the data were exported and analyzed using the Statistical Package for the Social Sciences^®^ (SPSS).

Descriptive statistics were performed, including absolute and relative frequencies for sociodemographic and professional data, as well as measures of central tendency and dispersion for (a) course satisfaction and (b) scores on the diagnostic (pre-course) and evaluative (post-course) activities. The Wilcoxon test with a 95% confidence interval assessed participant knowledge retention. The results are presented below.

## RESULTS

### First stage: Implementation of the course as an educational technology

To integrate the course into the Moodle platform, Articulate Storyline 3^®^ software was used. Although participants were unfamiliar with this program, its similarity to PowerPoint^®^ helped them understand its functionality. Interactive screens and custom layouts were developed within the tool, allowing participants to choose the content and the module with which to begin their studies. Migrating the educational materials, adjusting the software, and reviewing the course required 120 days (approximately 800 hours) of work by a four-member team. Upon completion, four SCORM learning objects (zipped packages) were created and customized in the VLE.

Customizing the virtual classroom required considerable team effort to organize and position the elements that guide and inform participants throughout the self-instructional course. The four SCORM packages were then uploaded to the virtual classroom, and their interactivity, screen transitions, and scoring functions were tested. This process ensured the course’s quality by correcting the inconsistencies identified by the team.

When accessing the virtual classroom, participants were welcomed by an introductory video for the multidisciplinary training course. The video provided basic navigation guidance and instructions on accessing the module content. Then, the character “Heloísa” introduced the course’s pedagogical structure, presenting the module’s name, objectives, and workload and guiding participants through the next steps of the learning journey.

**Figure 3:**
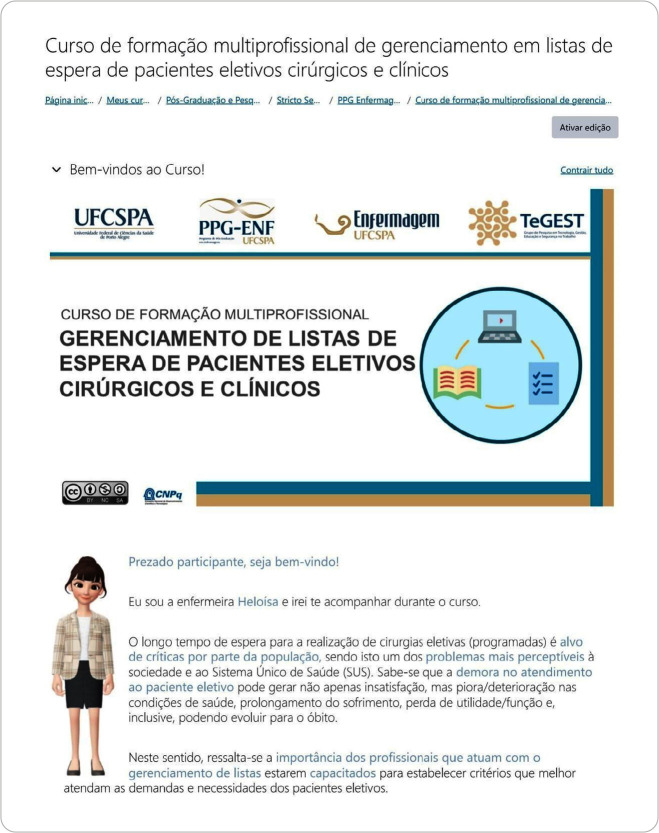
Course customization in the virtual learning environment. Porto Alegre, Rio Grande do Sul, Brazil, 2024

Participants were offered pedagogical and technical support via email and a social forum. Additionally, the platform displayed announcements with instructions for asynchronous contact with the course team, ensuring support at any time. The ICR was presented as a question, allowing participants to accept or decline the use of their course performance data for research purposes. All participants had access to the course modules (initially hidden), regardless of their ICR response. Figure 3 shows the initial course screen in the VLE.

The course’s social media outreach reached 8,882 accounts through Instagram^®^ feed posts and 690 accounts through stories. The main post received 248 likes and was shared 159 times. The course was also promoted on the websites of the Federal University of Health Sciences of Porto Alegre and the Federal Nursing Council. On institutional social media, it was shared by the university itself, the Regional Nursing Councils of Ceará and Tocantins, and the state chapters of the Brazilian Nursing Association in Maranhão, Santa Catarina, and Piauí.

### Second stage: Evaluation of participant satisfaction and knowledge retention in the multidisciplinary training course

A total of 768 registrations were received from healthcare professionals and students who enrolled in the VLE between January and March 2024. Of these, 429 (55.9%) accessed the course platform, with 255 (59.4%) completing the course and 174 (22.7%) dropping out.

The questionnaire designed to identify how participants learned about the course received 303 responses. The main sources reported were social media (122; 40.3%), email (72; 23.8%), websites (61; 20.1%), and referrals from supervisors, colleagues or work teams, and friends (48; 15.8%). Regarding geographic distribution, among the 307 participants who responded, most were from the Southern Region (159; 51.8%), followed by the Northeast (71; 23.1%), Southeast (33; 10.7%), North (25; 8.2%), and Central-West (19; 6.2%).

Table 1 presents participant satisfaction ratings regarding the course’s presentation, content, and applicability to professional practice. It also provides a profile of the 181 participants who completed the questionnaire available at the end of Module 4 and agreed to the ICR.

**Table 1:** Satisfaction evaluation and participant profile in the course (N = 181). Porto Alegre, Rio Grande do Sul, Brazil, 2024

Variables	n (%)
Course presentation	
Overall course presentation was appropriate	4.85 (0.45)^*^
Methods and tools used during the course were appropriate	4.79 (0.52)^*^
Diagnostic and evaluative activities were clear and coherent	4.85 (0.45)^*^
Rating assigned to course presentation	94.47 (9.38)^*^
Course content	
Course content was appropriate	4.85 (0.46)^*^
Course content met participants’ expectations	4.66 (0.67)^*^
Covered topics were appropriate	4.82 (0.54)^*^
Content sparked interest in the topic	4.82 (0.59)^*^
Rating assigned to course content	95.02 (9.55)^*^
Course applicability	
Knowledge was relevant to professional practice	4.66 (0.58)^*^
The course provided necessary and objective information for daily professional practice	4.73 (0.57)^*^
The course supported the development of professional competencies	4.80 (0.52)^*^
Rating assigned to course applicability	93.80 (11.27)^*^
Profile of participants who evaluated the course	
Sex (female)	147 (81.2)
Age (years)	35.81 (8.89)^*^
Brazilian regions	
South	97 (53.6)
Northeast	43 (23.8)
Southeast	20 (11)
North	14 (7.7)
Central-West	7 (3.9)
Experience working with waiting lists (years) (n=94)	1.83 (0.85-3.35)^†^

* *Mean (standard deviation); † Median (25th-75th percentile)*

Regarding the course presentation, 159 participants (87.8%) strongly agreed that the overall presentation was appropriate, and 19 (10.5%) somewhat agreed. However, 3 participants (1.7%) either somewhat disagreed or neither agreed nor disagreed. Similarly, 3 participants (1.7%) disagreed or remained neutral about the adequacy of the methods and tools used during the course, as well as the clarity and coherence of the diagnostic and evaluative activities.

Satisfaction with the course content exceeded 94%. Among those who selected “neither agreed nor disagreed,” “somewhat disagreed,” or “disagreed,” only 10 responses (5.6%) indicated that the content did not meet their expectations.

The applicability of the course to professional practice was considered relevant. However, 6 participants (3.4%) rated the content as only somewhat or minimally relevant. The same number of participants either neither agreed nor disagreed or somewhat disagreed that the course provided clear, objective, and applicable information for daily professional practice and the development of competencies in this field.

Among the participants who evaluated the course, 174 reported their educational attainment: 73 (41.9%) had completed undergraduate education, 61 (35.1%) were students, 33 (19%) held master’s degrees, and 7 (4%) held doctoral degrees. Of the 168 participants who responded about their institutional affiliation, 143 (85.1%) worked in public healthcare services.

The average score on the diagnostic activity (Module 1) was 76.25 ± 15.82 points, while the average score on the evaluative activity (Module 4) was 86.25 ± 16.26 points. Among the 255 course completers, 254 agreed to the ICR. Of these, 150 (58.9%) demonstrated knowledge retention by improving their post-course scores, 66 (25.9%) had lower scores after the course, and 38 (15.2%) scored the same on both the preand post-course activities.

**Figure 4:**
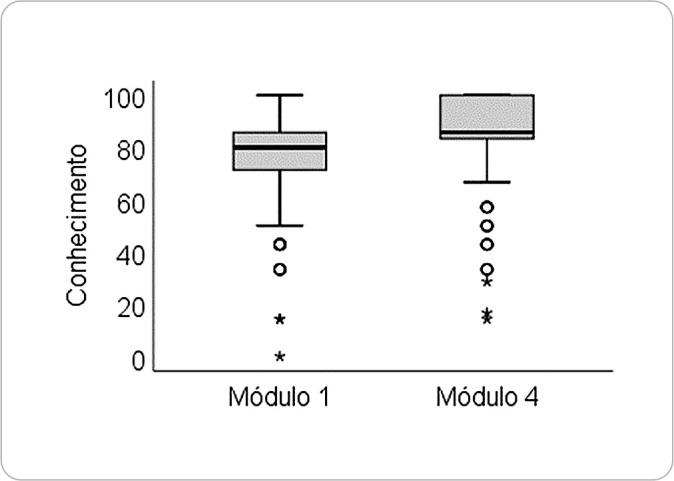
Knowledge retention in the course. Porto Alegre, Rio Grande do Sul, Brazil, 2024

The analysis showed significant knowledge retention when comparing diagnostic (pre-course) and evaluative (post-course) scores among the 254 participants (z = -8.09, p < 0.001), as shown in Figure 4, where the median score increased from 80 (71.4-85.1) to 85.7 (83.3-100).

## DISCUSSION

As a digital, free, multidisciplinary, and nationwide initiative, the course demonstrates broad potential for expanding access to knowledge for students and/or professionals involved in waiting list management. This was evidenced by the participation of individuals from different regions of the country.

Learning is no longer confined to the in-person formats of primary, secondary, or higher education; instead, it has become the primary means of professional training for those already in the workforce, particularly through continuing and permanent education. Considering remote education from the perspective of active participation involves embracing the collective challenge of building an educational environment among geographically dispersed individuals-one that enables interaction and promotes both individual and collective action supported by digital technologies^(11)^.

Professional education only becomes meaningful when it proves capable of generating effective and significant transformations in care practices and in the organization of work processes within healthcare services^(5)^. In hospital institutions, professional education must be developed on a continuous, active, and sustained basis, contributing to improving patient care^(12-14)^.

In this context, it is important to highlight that many healthcare institutions in Brazil are teaching hospitals, where medical residents are also part of the care team. Therefore, the annual turnover of residents is a natural occurrence. During these transition periods-for both residents and healthcare teams-a high number of errors related to waiting list management processes has been observed ^(5)^.

Even while recognizing the importance and contributions of medical residents to healthcare services^(15)^, educational interventions-such as the course discussed in this study-are also intended for this group and for all professionals involved in waiting list management. These tools play a fundamental role in enhancing service delivery by enabling professionals to contribute effectively to improving care and operational processes within their work institutions.

Given the importance of professional training and continuing education for the healthcare workforce, it is essential to implement educational initiatives that stimulate, support, and strengthen the ongoing development of healthcare workers. These actions aim to transform healthcare practices and uphold the core principles of Brazil’s Unified Health System (SUS)^(5,14)^. In this context, using technology in the professional education of healthcare workers should be considered as a means to support their training.

Notably, participants considered the course highly applicable to their professional practice, and the results regarding its effectiveness were positive. More than half of the participants demonstrated knowledge retention after completing the course. This finding supports the results of another study^(14)^, which showed that offering a self-instructional online multidisciplinary training course to healthcare professionals clearly promotes ongoing competency development and, consequently, contributes to improved performance in professional practice.

### Study limitations

A limitation of this study was the large number of participants who registered for or began the course but did not access the VLE or did not continue with the content.

### Contributions to the field of health and nursing

The course expanded healthcare professionals’ knowledge of waiting list management, potentially helping to reduce disparities and promote equity in patient care. Nursing is embracing this emerging area within healthcare service regulation, with an emphasis on improving care quality, enhancing patient safety, and aligning with the principles of SUS.

## CONCLUSIONS

The findings highlight the course’s positive impact on participants’ training, as reflected in significant knowledge retention and high satisfaction levels. This training is relevant to professional development and to improving public health service processes. The educational technology aligns with the Sustainable Development Goals (SDGs) by contributing to reducing disparities, decreasing population-level inequalities, and promoting equitable care and health outcomes for patients.

## Data Availability

The learning objects developed for the course are available on Padlet^®^: https://bit.ly/PainelComplexoRegulador.

## References

[1] Siciliani L, Hurst J. (2005). Tackling excessive waiting times for elective surgery: a comparative analysis of policies in 12 OECD countries. Health Policy.

[2] Lungu DA, Grillo RT, Nuti S. (2019). Decision making tools for managing waiting times and treatment rates in elective surgery. BMC Health Serv Res.

[3] Lisbôa RL, Kruel AJ, Marciniak JB, Paz AA. (2022). Actions of a regulatory nurse in the management of surgical waiting lists. Rev Bras Enferm.

[4] Lisbôa RL, Rodrigues CGS, Bittencourt CV, Paz AA. (2022). Estratégias de gerenciamento em listas de espera cirúrgicas: revisão integrativa. Acervo Saúde.

[5] Lisbôa RL, Furtado KF, Rosa VS, Evangelista CS, Paz AA. (2024). Educational technology for multidisciplinar training for managing waiting lists for elective patients. Rev Bras Enferm.

[6] Martins V, Santos E, Silva ÉFD. (2020). A educação online e os desenhos didáticos com interfaces móveis: autorias em ambientes virtuais de aprendizagem web e aplicativos. Debates Educ.

[7] Moreira JA, Schlemmer E. (2020). Por um novo conceito e paradigma de educação digital online. Rev UFG.

[8] Lisbôa RL. (2022). Curso de formação multiprofissional em gerenciamento de lista de espera de pacientes cirúrgicos e clínicos eletivos.

[9] Branch RM. (2009). Instructional design: the ADDIE approach.

[10] Santos MM, Mallon M, Boettcher S, Leonhardt GB, Vidal EF, Paz AA (2023). Avaliação de um curso de formação profissional para o cuidado de crianças com cateteres venosos centrais no domicílio. Rev Bras Inform Educ.

[11] Ministério da Saúde (BR) (2021). Ofício Circular nº 2/2021/CONEP/SECNS/MS - Orientações para procedimentos em pesquisas com qualquer etapa em ambiente virtual.

[12] Heckler V, Guidotti CDS. (2021). Ser professor no contexto online: processo formativo no ensino superior em tempos de pandemia. Debates Educ.

[13] Adamy KA, Zocche DAA, Vendruscolo C, Metelski FK, Argenta C, Valentini JS. (2018). Weaving permanent health education in the hospital contexto: experience report. RECOM.

[14] Corrêa CEC, Lopes GP, Silva CB, Paulin JN, Oliveira ND, Graeff MS (2022). Application of empathy map on educational actions carried out by nursing professionals. Rev Bras Enferm.

[15] Meschial WC, Ciccheto JRM, Lima MF, Menegaz JC, Echevarría-Guanilo ME, Oliveira MLF. (2021). Active teaching strategies improve nursing knowledge and skills to assist burn victims. Rev Bras Enferm.

[16] Blanco VM, Leonello VM, Souza CMS, Vasconcelos RO, Agreli HF. (2023). Health residency programs in a university hospital: a potent training setting for interprofessional collaborative practice. Interface.

