# Supplementary material for: Implementation and evaluation of a multidisciplinary training course in waiting list management
Source: Rev Bras Enferm. 2025 Dec 8;78(5):e20250142. doi: 10.1590/0034-7167-2025-0142 (PMC12707538; doi:10.1590/0034-7167-2025-0142)
Supplement: Translation

# Translation: Implementação e avaliação do curso de formação multiprofissional em gerenciamento de listas de espera

**DOI:** 10.1590/0034-7167-2025-0142pt

**Authors:** Rosa Ladi Lisbôa, Adriana Aparecida Paz

**Affiliations:** I Hospital Nossa Senhora da Conceição. Porto Alegre, Rio Grande do Sul, Brasil; II Universidade Federal de Ciências da Saúde de Porto Alegre. Porto Alegre, Rio Grande do Sul, Brasil

**Keywords:** Gestão em Saúde, Listas de Espera, Tecnologia Educacional, Tecnologia Aplicada às Listas de Espera, Enfermagem.

## Abstract

**Objetivo:**

Implementar e avaliar a satisfação e a retenção de conhecimentos em um curso multidisciplinar de formação em gerenciamento de listas de espera.

**Método:**

Estudo metodológico com duas etapas do modelo ADDIE - Design Instrucional, implementado em um ambiente virtual de aprendizagem (AVA) e executado entre outubro de 2023 e junho de 2024. Os dados foram coletados mediante questionários e relatórios do AVA, sendo submetidos à análise estatística descritiva e a teste de Wilcoxon (p<0,05).

**Resultados:**

Participaram 768 profissionais de saúde, dos quais 429 (55,9%) acessaram o AVA e 255 (59,4%) concluíram o curso. A avaliação do curso mostrou alta satisfação com a apresentação, conteúdo e aplicabilidade na prática profissional. A análise revelou retenção significativa de conhecimentos pelos participantes (z=-8,09, p<0,001).

**Conclusão:**

Os resultados evidenciam o impacto positivo do curso na formação dos participantes, refletido pela significativa retenção de conhecimento e elevada satisfação dos participantes.

## INTRODUÇÃO

A lista de espera de pacientes cirúrgicos e clínicos constitui um problema de abrangência nacional e gera impactos negativos às instituições de saúde e à sociedade, sobretudo ao paciente que aguarda pelo atendimento. A demora na realização dessas cirurgias potencializa uma série de problemas para os pacientes, tais como: piora/deterioração nas condições de saúde; prolongamento do sofrimento físico e psíquico; perda de utilidade/função corporal; e evolução para o óbito^(1,2)^. Há também os riscos econômicos, visto que pacientes com quadros clínicos mais graves tendem a permanecer mais tempo internados e, com isso, precisar de mais recursos, além de estarem mais expostos aos riscos de eventos adversos, como as infecções hospitalares, gerando um círculo vicioso de aumento de permanência e de riscos^(3,4)^.

Entre as fragilidades existentes nos sistemas de saúde, destacam-se o gerenciamento de listas de espera para cirurgias eletivas e a insuficiência de formação profissional nos serviços de saúde para a regulação de listas. Nesse sentido, salienta-se a importância da formação e educação continuada dos recursos humanos em saúde, o que é inclusive um princípio da Constituição brasileira^(5)^.

Por sua vez, o avanço do desenvolvimento tecnológico faz pensar que a aprendizagem não se restringe nem se efetiva somente dentro das instituições educativas, com atividades presenciais inseridas no cotidiano. Isso reforça a necessidade de criarem-se práticas pedagógicas capazes de ampliar a aprendizagem e o ensino no ciberespaço, com percursos, contextos e sujeitos diferenciados^(6)^.

Além disso, o ensino à distância (EaD) na capacitação de profissionais enfatiza a construção e a socialização do conhecimento, operacionalizando os princípios e fins da educação. Dessa forma, independentemente do tempo e do lugar, qualquer pessoa pode tornar-se agente de sua aprendizagem graças ao uso de materiais diferenciados e meios de comunicação que permitam a interatividade (síncrona ou assíncrona) e o trabalho colaborativo/cooperativo^(7)^.

Este estudo baseia-se na dissertação de mestrado profissional que criou uma tecnologia educativa construída, avaliada e implementada com rigor técnico-científico. Essa tecnologia enquadra-se na modalidade de curso de formação, visando qualificar os profissionais da saúde e técnicos administrativos que atuam em serviços de regulação de listas de espera de pacientes cirúrgicos e clínicos eletivos^(5)^.

A otimização do gerenciamento de listas de espera de pacientes cirúrgicos eletivos é essencial para o funcionamento do Sistema Único de Saúde (SUS). Assim, essa tecnologia mostra-se relevante por contribuir para a capacitação das diversas categorias atuantes na gestão dessas listas de espera.

## OBJETIVO

Implementar e avaliar a satisfação e a retenção de conhecimentos em um curso multidisciplinar de formação em gerenciamento de listas de espera.

## MÉTODOS

### Aspectos éticos

Os dados analisados neste estudo são provenientes de uma dissertação de mestrado^(8)^. O presente estudo foi aprovado pelo Comitê de Ética em Pesquisa, atendendo às diretrizes e normas regulamentadoras para pesquisas com seres humanos. O Termo de Consentimento Livre e Esclarecido foi obtido de todos os participantes que formalizaram a aceitação no Registro de Consentimento Livre e Esclarecido (RCLE). Nesse documento, foram assegurados o anonimato, a confidencialidade, a privacidade e o sigilo das informações. O projeto obteve a aprovação do Comitê de Ética em Pesquisa sob parecer nº 5.103.774/2021.

### Desenho, período e local do estudo

Este estudo metodológico foi desenvolvido em cinco etapas, com base no Design Instrucional do modelo ADDIE (acrônimo de Análise, Desenho, Desenvolvimento, Implementação e Avaliação)^(9)^. O modelo é dividido em duas fases: concepção e execução. A fase de concepção (Análise, Desenho e Desenvolvimento) compreendeu a construção da tecnologia educativa na modalidade de curso de formação multiprofissional. Ainda durante essa fase, foram realizadas a análise de demanda, o desenho pedagógico do curso e o desenvolvimento de conteúdo em *storyboards* com a subsequente avaliação de adequação do conteúdo por um comitê de especialistas, sendo que essa fase da pesquisa foi publicada recentemente^(5)^. A fase de execução abrangeu as etapas de implementação do curso em ambiente virtual de aprendizagem (AVA) bem como avaliação de satisfação e retenção de conhecimento do curso pelos participantes. Essas duas últimas etapas concluíram a aplicação do modelo ADDIE, foco deste estudo.

O produto foi desenvolvido e implementado no AVA em alinhamento com um Programa de Pós-Graduação em Enfermagem - Mestrado Profissional de uma universidade pública do sul do Brasil. A equipe executora incluiu duas enfermeiras (uma doutora e uma mestra) e duas estudantes de enfermagem (bolsistas de iniciação científica e iniciação à docência). As etapas ocorreram entre outubro de 2023 e junho de 2024.

### Procedimentos metodológicos

#### Primeira etapa: Implementação do curso como uma tecnologia educativa

A implementação se deu após a fase de concepção do curso no modelo ADDIE. Os *storyboards* desenvolvidos na ferramenta PowerPoint^®^ foram exportados para o software Articulate Storyline 3^®^. Essa ferramenta permitiu o desenvolvimento da forma pela qual o conteúdo é exibido na sala virtual, contando com vários recursos gráficos, responsividade ao tamanho e resolução de tela. Além disso, fornece soluções otimizadas para telas *touch*, tecnologia presente em dispositivos móveis.

O Sharable Content Object Reference Model (SCORM) é um pacote de arquivos gerado com a exportação no formato nativo do Articulate Storyline 3^®^ (*.STORY). O SCORM contém informações e parâmetros técnicos que podem ser operados pelo *Learning Management Systems* (LMS, traduzido para a língua portuguesa como Sistemas de Gerenciamento de Aprendizagem) no AVA. O formato SCORM é um padrão intercambiável no ensino à distância que permite implementar o conteúdo no LMS, conforme ilustra a Figura 1.

**Figura 1:** SCORM do curso. Porto Alegre, Rio Grande do Sul, Brasil, 2024

A sala virtual no AVA foi disponibilizada pelo Núcleo de Educação à Distância e Núcleo da Tecnologia e Informação da Universidade, o que permitiu estruturar e organizar os objetos de aprendizagem na sala virtual (https://moodle.ufcspa.edu.br/course/view.php?id=2768). Para estruturar essa sala, foram desenvolvidos layouts, textos e vídeos instrucionais, customizados com a identidade visual construída nos *storyboards* para facilitar o acesso e adesão dos participantes ao curso.

O cadastro do curso ocorreu no Sistema Único de Registros (SIUR) da universidade, em dezembro de 2023, para apreciação pela Comissão dos Programas de Pós-Graduação. Foi aprovada a oferta de mil vagas para a comunidade interna da instituição e participantes externos (https://bit.ly/GestaoLeitosPPGEnf), com inscrições entre dezembro de 2023 e março de 2024.

A divulgação foi feita nas mídias sociais do Grupo de Pesquisa em Tecnologia, Gestão, Educação e Segurança no Trabalho (TeGEST) do Programa de Pós-Graduação em Enfermagem da universidade bem como nos perfis do Conselho Federal de Enfermagem e Conselhos Regionais de Enfermagem. Também foi divulgado via e-mail, com envio do convite e ficha de inscrição às instituições de ensino superior, instituições de saúde, conselhos profissionais e associações de enfermagem e médicas. O curso ocorreu em modalidade EaD e autoinstrucional entre janeiro e abril de 2024 e teve carga horária de 20 horas.

#### Segunda etapa: Avaliação de satisfação e retenção de conhecimentos dos participantes do curso multiprofissional

Trata-se de um estudo exploratório-descritivo, com abordagem quantitativa. O cenário de estudo foi o “Curso de formação multiprofissional de gerenciamento em listas de espera de pacientes eletivos cirúrgicos e clínicos” (Figura 2), com carga horária de 20 horas e disponibilização no AVA da instituição.

**Figura 2:** Curso de formação multiprofissional no gerenciamento de listas de espera de pacientes eletivos cirúrgicos e clínicos. Porto Alegre, Rio Grande do Sul, Brasil, 2024

A estrutura dos Módulos 1 e 4 é semelhante. O primeiro continha uma atividade diagnóstica (antes do conteúdo) intitulada “Conhecimentos prévios sobre gerenciamento das listas de espera de pacientes eletivos”, e o último apresentava uma atividade avaliativa (depois do conteúdo) denominada “Avaliação da aprendizagem sobre gerenciamento de listas de espera de pacientes eletivos”. Cada um dos módulos possuía uma carga horária de uma hora para a resolução de três casos clínicos, contendo sete questões de escolha simples, com quatro alternativas cada.

Por meio de cliques, o participante pode escolher qual caso clínico realizar de acordo com o seu interesse. Utilizaram-se diferentes estratégias na composição das questões para diversificar sua exigência e complexidade. Esses dois módulos diferem no ordenamento dos casos clínicos, nos nomes fictícios, na localização geográfica, no ordenamento das respostas corretas das perguntas e no feedback ao final de cada questão, o qual ocorreu apenas na atividade avaliativa.

No total, 428 participantes aceitaram participar da avaliação de satisfação com o curso (apresentação, conteúdo e aplicabilidade), realizada por meio de um questionário cujo endereço eletrônico foi disponibilizado no último módulo. Eles também registraram o consentimento livre e esclarecido disponibilizado no AVA. Este estudo incluiu os participantes que concluíram os quatro módulos do curso - permitindo a visualização total do conteúdo e da interatividade proporcionada pelo EaD - e que responderam ao questionário de avaliação. Depois de formalizar a aceitação no RCLE, foi disponibilizado outro questionário para coleta de informações sobre a divulgação do curso em canais de comunicação e procedência dos participantes. Os dados relacionados à avaliação da retenção de conhecimento referente aos Módulos 1 e 4 realizados pelo participante foram extraídos do AVA.

A coleta de dados deste estudo ocorreu exclusivamente no banco de dados de dois formulários editados no Google Forms^®^ e no relatório dos participantes gerado pelo AVA. Dados referentes ao número de inscritos, evadidos e concluintes foram coletados no AVA por meio de um instrumento em formato de tabela, denominado de “relatório de extração de dados”.

Em junho de 2024, os dados de avaliação de satisfação com o curso foram extraídos do banco de dados do Google Forms^®^ dos participantes que responderam ao questionário “Avaliação do curso de formação profissional”.

O questionário para avaliar a satisfação dos participantes continha 26 questões (múltipla escolha, única escolha aberta e de escala numérica). Foi estruturado em quatro seções para avaliação do curso: a) Apresentação; b) Conteúdo; c) Aplicabilidade; e d) Caracterização sociodemográfica e laboral dos participantes. Essas questões abordavam a qualidade dos métodos e ferramentas tecnológicas utilizadas, a clareza e coerência das questões avaliativas, a adequação do conteúdo e linguagem, a aplicabilidade e relevância para a prática profissional, bem como a capacidade do curso de despertar interesse e corresponder às expectativas. Esses aspectos foram avaliados por meio de perguntas dicotômicas (sim ou não), escala de Likert de cinco pontos e escala numérica de 0 a 100 pontos, elaboradas com base em outro estudo^(10)^ e adaptadas pelos autores.

### Análise dos resultados e estatística

Os dados foram extraídos de um banco de dados do AVA em planilha do Microsoft Excel^®^ referente à aceitação em participar do estudo e obtidos do formulário do Google Forms^®^ em planilha do Google Sheets^®^. Foram salvos em um computador local no formato de planilha Excel^®^ da Microsoft^®^ para tratamento dos dados, de modo que o dado sensível fosse protegido e impossibilitasse a identificação do participante^(11)^. O banco de dados continha apenas as variáveis de interesse deste estudo, e os dados foram exportados e analisados no programa Statistical Package for the Social Sciences^®^ (SPSS).

Realizou-se análise descritiva com frequências absoluta/relativa para dados sociodemográficos e laborais, além de medidas de tendência central e dispersão para: (a) satisfação com o curso; e (b) pontuação nas atividades diagnóstica (pré-curso) e avaliativa (pós-curso). Para avaliar a retenção de conhecimento pelos participantes, utilizou-se o teste de Wilcoxon, com intervalo de confiança de 95%. Os resultados são apresentados a seguir.

## RESULTADOS

### Primeira etapa: Implementação do curso como uma tecnologia educativa

Para integrar o curso na plataforma Moodle, foi necessário utilizar o software Articulate Storyline 3^®^. Embora os participantes não tivessem familiaridade com esse programa, sua semelhança com o Power Point^®^ facilitou a compreensão do funcionamento. Nele, foram implementados layouts e telas interativas em que o participante pode escolher o conteúdo e o módulo pelo qual deseja iniciar os estudos. A migração dos materiais didáticos, os ajustes no software e a checagem do curso demandaram 120 dias (cerca de 800 horas) de trabalho de quatro membros da equipe. Após a finalização, foram obtidos quatro objetos de aprendizagem em formato SCORM (pacotes zipados), customizados no AVA.

A customização da sala virtual exigiu empenho da equipe na organização e disposição dos elementos que orientam e informam o participante no decorrer de um curso autoinstrucional. Em seguida, iniciou-se a transferência dos quatro SCORM para a sala virtual, testando sua interatividade, transição de telas e pontuação nas diferentes atividades. Esse procedimento garantiu a qualidade do curso ao corrigir as inconsistências identificadas pela equipe.

Ao acessar a sala virtual, o participante é recebido por um vídeo de boas-vindas do curso de formação multiprofissional. Esse material apresenta as orientações básicas sobre a navegação na plataforma e acesso aos conteúdos dos módulos. Em seguida, a personagem “Heloísa” introduz a matriz pedagógica do curso, detalhando o nome, os objetivos e a carga horária de cada módulo, além de conduzir o participante pelos próximos passos da jornada de aprendizagem.

Foram disponibilizados aos participantes o acesso ao suporte pedagógico e técnico por meio de endereços de e-mail e de um fórum social. Além disso, a plataforma exibia avisos com instruções para contato assíncrono com a equipe responsável, garantindo suporte a qualquer momento. O RCLE foi elaborado em formato de questão, permitindo ao participante aceitar ou recusar a utilização de seus dados de desempenho no curso para fins de pesquisa. Todos os participantes tinham acesso aos módulos do curso (inicialmente ocultos), independentemente de sua escolha no RCLE. A seguir, a Figura 3 apresenta a tela inicial do AVA do curso.

**Figura 3:** Customização do curso no ambiente virtual de aprendizagem. Porto Alegre, Rio Grande do Sul, Brasil, 2024

A divulgação do curso nas mídias sociais alcançou 8.882 contas nas publicações no *feed* do Instagram^®^ e 690 contas nos *stories*. O post teve 248 curtidas e 159 compartilhamentos. O curso foi divulgado no website da Universidade Federal de Ciências da Saúde de Porto Alegre e do Conselho Federal de Enfermagem. Nas redes sociais de instituições, houve a divulgação pela referida universidade, pelos Conselhos Regionais de Enfermagem do Ceará e Tocantins e pelas representações estaduais da Associação Brasileira de Enfermagem no Maranhão, Santa Catarina e Piauí.

### Segunda etapa: Avaliação de satisfação e retenção de conhecimentos dos participantes do curso multiprofissional

Foram obtidas 768 inscrições entre profissionais e estudantes da área da saúde cadastrados no AVA entre janeiro e março de 2024. Do total, 429 (55,9%) acessaram a plataforma do curso, com 255 (59,4%) concluintes e 174 (22,7%) evadidos.

O questionário para conhecer o canal de divulgação do curso obteve 303 respostas, destacando-se como principais fontes as redes sociais (122; 40,3%), e-mail (72; 23,8%), websites (61; 20,1%) e indicações de chefias, colegas/grupo de trabalho e amigos (48; 15,8%). Quanto à procedência dos 307 participantes, a maioria era da Região Sul (159; 51,8%), seguida da Região Nordeste (71; 23,1%), Região Sudeste (33; 10,7%), Região Norte (25; 8,2%) e Região Centro Oeste (19; 6,2%).

A Tabela 1 apresenta a avaliação da satisfação com a apresentação do curso, conteúdo e aplicabilidade na prática profissional e exibe a caracterização de 181 participantes que responderam ao questionário disponibilizado ao final do Módulo 4 do curso e manifestaram a concordância com o RCLE

**Tabela 1:** Avaliação da satisfação e caracterização dos participantes do curso (N = 181). Porto Alegre, Rio Grande do Sul, Brasil, 2024

Variáveis	n (%)
Apresentação do curso	
Apresentação geral do curso adequada	4,85 (0,45)^*^
Métodos e ferramentas utilizados durante o curso eram adequados	4,79 (0,52)^*^
As atividades diagnóstica e avaliativa eram claras e coerentes	4,85 (0,45)^*^
Valor atribuído à apresentação do curso	94,47 (9,38)^*^
Conteúdo do curso	
Conteúdo do curso adequado	4,85 (0,46)^*^
Conteúdo do curso correspondeu às suas expectativas	4,66 (0,67)^*^
Conteúdos abordados foram adequados	4,82 (0,54)^*^
Conteúdo desperta o interesse para o tema	4,82 (0,59)^*^
Valor atribuído ao conteúdo do curso	95,02 (9,55)^*^
Aplicabilidade do curso	
Conhecimentos foram relevantes para a prática profissional	4,66 (0,58)^*^
Curso ofereceu informações necessárias e objetivas para a prática profissional diária	4,73 (0,57)^*^
Curso proporcionou o desenvolvimento de competências para a prática profissional	4,80 (0,52)^*^
Valor atribuído da aplicabilidade do curso	93,80 (11,27)^*^
Caracterização dos participantes que avaliaram o curso	
Sexo (feminino)	147 (81,2)
Idade (anos)	35,81 (8,89)^*^
Regiões brasileiras	
Sul	97 (53,6)
Nordeste	43 (23,8)
Sudeste	20 (11)
Norte	14 (7,7)
Centro-Oeste	7 (3,9)
Atuação em listas de espera em anos (n=94)	1,83 (0,85-3,35)^†^

* Média (desvio-padrão);

† Mediana (percentil 25-percentil 75)

Sobre a apresentação curso, 159 participantes (87,8%) concordaram totalmente que a apresentação geral do curso era adequada, e 19 (10,5%) concordaram parcialmente; entretanto, 3 (1,7%) discordaram parcialmente dessa afirmativa ou não concordaram nem discordaram. Igualmente, 3 (1,7%) mostraram-se discordantes ou neutros sobre os métodos e ferramentas utilizados durante o curso serem adequados e sobre as atividades diagnóstica e avaliativa serem claras e coerentes.

A satisfação com o conteúdo foi superior a 94%. Entre aqueles que escolheram a opção “não discordo” e “não concordo” e “discordo parcialmente” sobre o conteúdo, apenas 10 (5,6%) respostas reforçam que o conteúdo não correspondeu às expectativas.

A aplicabilidade do curso na prática profissional foi considerada relevante, entretanto apenas 6 (3,4%) consideraram como razoavelmente relevantes ou pouco relevantes os conhecimentos do curso na prática profissional. Esse mesmo número de participantes não concorda e não discorda ou discorda parcialmente que o curso ofereceu informações necessárias e objetivas para a aplicação na prática profissional diária e desenvolvimento de competências nessa temática.

Dos participantes que avaliaram o curso, 174 responderam sobre o seu nível de formação. Eram 73 (41,9%) graduados, 61 (35,1%) estudantes, 33 (19%) mestres e 7 (4%) doutores. Dos 168 que responderam sobre a natureza da instituição, 143 (85,1%) trabalhavam em serviços públicos.

A média de pontuação da atividade diagnóstica (Módulo 1) foi de 76,25±15,82 pontos, enquanto a média da atividade avaliativa (Módulo 4) foi de 86,25±16,26 pontos. Dos 255 concluintes do curso, 254 concordaram com o RCLE, dentre os quais 150 (58,9%) demonstraram alcançar a retenção de conhecimento com a realização do curso, 66 (25,9%) reduziram sua pontuação inicial, e 38 (15,2%) permaneceram com o mesmo valor obtido em ambas as atividades realizadas (antes e depois) do curso.

A análise revelou retenção significativa de conhecimentos considerando-se a atividade diagnóstica (antes) e avaliativa (depois) do curso para os 254 participantes (z = -8,09, p < 0,001), conforme mostra a Figura 4, em que a mediana passa de 80 (71,4-85,1) pontos para 85,7 (83,3-100).

**Figura 4:** Retenção de conhecimentos no curso. Porto Alegre, Rio Grande do Sul, Brasil, 2024

## DISCUSSÃO

Por ser digital, gratuito, multiprofissional e de alcance nacional, o curso evidencia a ampla possibilidade de acesso ao conhecimento para os estudantes e/ou profissionais que atuam no gerenciamento das listas de espera. Isso foi evidenciado pelo fato de os participantes do curso serem de diferentes regiões do país.

A aprendizagem não se limita à natureza presencial do ensino fundamental, médio ou universitário; entretanto, é o modo pelo qual tem sido realizada a formação profissional dos que estão inseridos no mercado de trabalho sob o formato de educação permanente e continuada. Pensar o ensino remoto sob a perspectiva da participação ativa é assumir o desafio coletivo de construir um cenário educativo com pessoas geograficamente distantes, a fim de oportunizar a interação e promover ações individuais e coletivas com o auxílio das tecnologias digitais^(12)^.

A educação profissional só adquire sentido quando demonstra capacidade de promover transformações efetivas e significativas nas práticas assistenciais e na organização dos processos de trabalho dos serviços de saúde^(5)^. A educação profissional nas instituições hospitalares deve ser desenvolvida de maneira permanente, ativa e consolidada, contribuindo na qualificação da assistência ao paciente^(13-15)^.

Nesse sentido, cabe ressaltar que várias instituições de saúde brasileiras são hospital-escola, ou seja, os profissionais que estão cursando a residência também integram a equipe de trabalho, sendo natural a rotatividade de profissionais residentes anualmente. Nesse período de adaptação para os profissionais e para as equipes, percebe-se elevado número de erros relativos ao processo de gerenciamento de listas de espera^(5)^.

Mesmo entendendo-se a importância e a contribuição dos residentes no serviço de saúde^(16)^, ações educativas - como o curso abordado neste estudo - destinam-se também a tal público e a todos os profissionais que atuam no gerenciamento de listas de espera. Dessa forma, essas ferramentas cumprem um papel fundamental ao qualificar o atendimento às demandas do serviço, permitindo que esses profissionais colaborem de maneira efetiva para a melhoria dos atendimentos e dos processos em curso nas instituições em que atuam.

Tendo em vista a importância da formação e educação continuada dos recursos humanos em saúde, é imprescindível realizar ações educativas para estimular, acompanhar e fortalecer a qualificação profissional dos trabalhadores que atuam na área da saúde, visando à transformação das práticas de saúde e ao atendimento dos princípios fundamentais do SUS^(5,15)^. Nesse sentido, deve-se conjecturar o uso da tecnologia na educação profissional dos trabalhadores da saúde para favorecer sua formação profissional.

Destaca-se que a aplicabilidade do curso na prática profissional foi considerada relevante entre os participantes, e os resultados referentes à sua efetividade mostram-se positivos quanto à utilização dessa tecnologia no âmbito educativo, já que mais da metade dos participantes apresentou retenção de conhecimentos sobre a temática após sua finalização. Esse achado corrobora dados de um estudo^(15)^ no qual se mostrou que a oferta do curso de formação multiprofissional autoinstrucional on-line aos trabalhadores atuantes na área da saúde promove, indubitavelmente, a oportunidade para o constante aperfeiçoamento das competências e, consequentemente, propicia melhor desempenho nas práticas profissionais.

### Limitações do estudo

Apresenta-se como limitação o número elevado de participantes que se inscreveram ou iniciaram o curso, porém não acessaram o AVA ou não deram continuidade.

### Contribuições para a área da saúde e da enfermagem

A oferta do curso ampliou os conhecimentos dos profissionais na gestão da lista de espera, potencialmente mitigando disparidades e promovendo equidade no atendimento ao paciente. A enfermagem assume essa nova área de atuação na regulação dos serviços de saúde, com foco na qualificação assistencial, segurança do paciente e alinhamento com os princípios do SUS.

## CONCLUSÕES

Os resultados mostram o impacto positivo do curso na formação dos participantes, evidenciado pela significativa retenção de conhecimento e elevada satisfação dos participantes. Tal formação é relevante para o desenvolvimento profissional e melhoria dos processos de saúde pública. Essa tecnologia educacional coaduna com os Objetivos de Desenvolvimento Sustentável (ODS) na busca por amenizar as diferenças, reduzir as desigualdades entre a população bem como promover atendimento justo e saúde ao paciente.

## Data Availability

Os objetos de aprendizagem do curso estão disponíveis no Padlet^®^ (https://bit.ly/PainelComplexoRegulador).
